# Effects of 7 days on an ad libitum low-fat vegan diet: the McDougall Program cohort

**DOI:** 10.1186/1475-2891-13-99

**Published:** 2014-10-14

**Authors:** John McDougall, Laurie E Thomas, Craig McDougall, Gavin Moloney, Bradley Saul, John S Finnell, Kelly Richardson, Katelin Mae Petersen

**Affiliations:** Dr. McDougall’s Health and Medical Center, PO Box 14039, Santa Rosa, CA 95402 USA; Madison, NJ USA; Healthy Living Program, Northwest Permanente, Beaverton, OR USA; Department of Biostatistics, University of North Carolina, Chapel Hill, NC USA; AOMA Graduate School of Integrative Medicine, Austin, TX USA; Richardson Statistical Consulting, Iowa City, IA USA

**Keywords:** Low-fat diet, Vegan diet, Vegetarian diet, Hypertension, Cholesterol, Hyperlipidemia, Type 2 diabetes, Weight loss, Heart disease

## Abstract

**Background:**

Epidemiologic evidence, reinforced by clinical and laboratory studies, shows that the rich Western diet is the major underlying cause of death and disability (e.g, from cardiovascular disease and type 2 diabetes) in Western industrialized societies. The objective of this study is to document the effects that eating a low-fat (≤10% of calories), high-carbohydrate (~80% of calories), moderate-sodium, purely plant-based diet ad libitum for 7 days can have on the biomarkers of cardiovascular disease and type 2 diabetes.

**Methods:**

Retrospective analysis of measurements of weight, blood pressure, blood sugar, and blood lipids and estimation of cardiovascular disease risk at baseline and day 7 from 1615 participants in a 10-day residential dietary intervention program from 2002 to 2011. Wilcoxon’s signed-rank test was used for testing the significance of changes from baseline.

**Results:**

The median (interquartile range, IQR) weight loss was 1.4 (1.8) kg (p < .001). The median (IQR) decrease in total cholesterol was 22 (29) mg/dL (p < .001). Even though most antihypertensive and antihyperglycemic medications were reduced or discontinued at baseline, systolic blood pressure decreased by a median (IQR) of 8 (18) mm Hg (p < .001), diastolic blood pressure by a median (IQR) of 4 (10) mm Hg (p < .001), and blood glucose by a median (IQR) of 3 (11) mg/dL (p < .001). For patients whose risk of a cardiovascular event within 10 years was >7.5% at baseline, the risk dropped to 5.5% (>27%) at day 7 (p < .001).

**Conclusions:**

A low-fat, starch-based, vegan diet eaten ad libitum for 7 days results in significant favorable changes in commonly tested biomarkers that are used to predict future risks for cardiovascular disease and metabolic diseases.

## Introduction

The primary goal of health care should be to decrease all-cause morbidity and mortality. In industrialized societies including those in the United States and Europe, the chief causes of death and disability are noninfectious chronic diseases: atherosclerotic vascular disease, epithelial cell cancers, type 2 diabetes, and autoimmune disorders [1]. The leading underlying cause of these diseases is the rich Western diet, with its emphasis on animal-source foods (i.e., meat, fish, eggs, and dairy foods) and fat-laden and sugary processed foods [2, 3]. These diseases are becoming increasingly prevalent in newly industrialized countries in Central America, South America, and Asia as they, too, adopt Western eating styles [4].

The burden of Western disease can be dramatically reduced by eliminating animal-source foods and vegetable fats from the diet and replacing those foods with low-fat, plant-based foods. When a food rationing system during World War I severely restricted the Danish population’s intake of meats, dairy products, fats, and alcohol but placed no restrictions on such foods as barley, bread, potatoes, and vegetables, Denmark achieved the lowest mortality rate in its history [5]. Similarly, the mortality due to diabetes in England and Wales decreased sharply while wartime food rationing in both World War I and World War II limited access to animal-source foods and fats, only to increase after the those foods became available again in peacetime [6].

A physician can help his or her patients achieve similar benefits by teaching them to eat a low-fat (≤10% of calories), high-fiber, high-carbohydrate (~80% of calories), purely plant-based (vegan) diet [7–14]. The goal of the present study is to document the improvements in biomarkers of the risk of cardiovascular and metabolic diseases that can be achieved within 7 days when patients are allowed to eat such a diet to satiety under medical supervision in an inpatient setting.

## Methods

This study is a retrospective analysis of the records of patients who attended a physician-supervised 10-day residential program (the McDougall Program) from 2002 to 2011. This ongoing program used Internet marketing to attract patients from a wide geographic area to spend 10 days at a hotel in Santa Rosa, California, where they received dietary counseling and were fed a low-fat (≤10% of calories) diet based on minimally refined plant foods ad libitum to satiety. The educational staff included a medical doctor, a registered dietitian, a psychologist, exercise coaches, and cooking instructors. Patients were also given opportunities for light to moderate exercise. No stress reduction techniques or meditations were included in the program.

### Medical workup

Upon admission, a standardized questionnaire was used to ask patients if they had any history of hypertension, coronary artery disease, diabetes, hypothyroidism, multiple sclerosis, hypercholesterolemia, or overweight. The patient’s age, sex, and ethnicity were also recorded.

A licensed medical doctor saw all patients on at least 3 occasions during the program, and blood pressure was recorded daily. At baseline, the physician took the patient’s history and performed a physical examination. The physician then recommended appropriate changes to each patient’s medications. Medications for hypertension and diabetes were reduced or discontinued at baseline in order to lessen the risk of hypotension and hypoglycemia. Cholesterol-lowering medications (statins) were continued throughout the program for those people taking this class of medication upon entering the program.

To facilitate analysis, the following data were recorded according to standardized methods at baseline and on day 7: weight, systolic and diastolic blood pressure, total cholesterol, triglycerides, glucose, blood urea nitrogen, and creatinine. After August 2006, HDL and LDL cholesterol were also measured at baseline and day 7. Body weights were measured with a Detecto® 6800 portable bariatric scale. Blood pressure readings were obtained by a trained operator, manually and/or using an Omron® professional sphygmomanometer. Blood tests were analyzed by using standard medical hospital laboratory procedures.

### Menu design

The hotel’s kitchen staff prepared foods according to prescribed guidelines. No animal-derived ingredients (e.g, meat, fish, eggs, or dairy products) and no isolated vegetable oils (e.g, olive, corn, safflower, flaxseed, or rapeseed oil) were used. Meals were based around common starches, including wheat flour products, corn, rice, oats, barley, quinoa, potatoes, sweet potatoes, beans, peas, and lentils, with the addition of fresh fruits and non-starchy green, orange, and yellow vegetables. The macronutrient profile was roughly 7% fat, 12% protein, and 81% carbohydrate by calorie. Meals were served buffet-style, and participants were encouraged to eat to the full satisfaction of their appetite.

To ensure that the foods would be acceptable to palates accustomed to Western diets, small amounts of simple sugars, salt, and various spices were used in meal preparation. Sugar was included in condiment sauces (e.g, ketchup and barbecue sauce) and in the low-fat desserts that were served after evening meals. Patients could also add sugar to their cereal in the morning.

The kitchen staff used minimal salt, mostly in the form of soy sauce, when preparing the meals. The basic meal plan provided roughly 1000 mg of sodium per day. However, saltshakers were provided at meals, and participants were allowed to use regular table salt ad libitum on the surface of their foods for taste. We conclude that even if a patient added a total of a half-teaspoon of salt per day, the diet would still qualify as low-sodium (approximately 2 g of sodium daily).

### Statistical methods

Data from patients who attended the program from 2002 to 2011 were analyzed in 2014. For patients who attended the program more than once, each visit was regarded as a separate patient record. Records from subjects under the age of 21 were excluded from the analysis. Records with missing data for baseline or day 7 were also excluded.

Before August 2006, tests for HDL and LDL cholesterol were not routinely ordered. Only the records that had baseline and day 7 values were included in the analysis of HDL and LDL. The average total cholesterol values and the change in total cholesterol from day 1 to day 7 for the patients with missing HDL and LDL data were compared to the respective values for the patients with complete data on HDL and LDL.

The change from baseline in biomarkers was analyzed for risk categories based on the patients’ baseline variables. The cutoffs for the risk categories were arbitrary: total cholesterol <150, 150–179, 180–209, 210–239, or ≥240 mg/dL; triglycerides <150, 150–199, 200–499, or ≥500 mg/dL; glucose <100, 100–126, or >126 mg/dL; systolic blood pressure < 140 or ≥140 mm Hg; diastolic blood pressure <90 or ≥90 mm Hg; creatinine <1, 1–1.4, or >1.4 mg/dL; and blood urea nitrogen <10, 10–20, or >20 mg/dL. Because the patients’ height was not recorded, the patients could not be stratified according to body mass index. Instead, they were stratified according to their weight, as expressed in standard deviations from the mean for his or her sex.

Because none of the continuous biomarker variables were normally distributed, Wilcoxon’s signed-rank tests were used to compare baseline values to day 7 values for all biomarker variables. Each comparison was evaluated for the baseline risk categories, as described above. McNemar’s test for paired proportions was conducted to compare the percentage of patients with elevated biomarkers (total cholesterol ≥200 mg/dL, SBP ≥140 mm Hg and DBP ≥90 mm Hg) at baseline whose values fell to normal at day 7 to the percentage of patients with normal biomarkers at baseline whose values were considered elevated at day 7.

In 2013, the American College of Cardiology and American Heart Association published guidelines for estimating a patient’s probability of having a cardiac health event (atherosclerotic cardiovascular disease, ASCVD) within 10 years [15]. The guidelines define high ASCVD as a risk >7.5%. The ASCVD risk can be calculated only for patients whose records include all of the relevant variables: age, total cholesterol, HDL cholesterol, systolic blood pressure, smoking status, diabetes diagnosis, and treatment for high blood pressure. For those patients, the ASCVD risks were calculated twice: once for the baseline values and again for the day 7 values. All of the patients who were on blood pressure medication at baseline were considered to be under treatment for high blood pressure at day 7 as well.

All statistical analyses were conducted by using SAS 9.3 (Cary, NC).

## Results

A total of 1703 patients attended the program from 2002 to 2011. Of these, 46 patients went through the program more than once. The records for the 12 patients under the age of 21 were excluded. As a result, a total of 1615 patient-records were included in the analyses. There were 1092 records with baseline and day 7 records for HDL and 1091 with baseline and day 7 records for LDL.

Table 1 shows that the majority of patients were female (65.1%), white non-Hispanic (92.1%), and with a median (IQR) age of 58 (18) years. Upon admission, 84.3% of the participants self-reported some history of disease. Nearly 64% reported a history of hypercholesterolemia, and 39.6% reported a history of hypertension. Most of the patients (79.5%) reported being on at least 1 medication at baseline for diabetes, hypertension, or elevated cholesterol. Additionally, the majority of the sample self-reported as being overweight (71.6%).

**Table 1:** **Demographic variables for full sample (N = 1615)**

Variable	Value
Sex, no. (%)	
Male	563 (34.9)
Female	1052 (65.1)
Race/ethnicity, no. (%)	
Asian	32 (2.0)
Black	45 (2.8)
Latino	36 (2.2)
White	1488 (92.1)
Other	14 (0.9)
Age, median (IQR)	58 (18)
< 50 years, no. (%)	459(28.4)
50 to 64 years, no. (%)	689 (42.7)
65 or older years, no. (%)	467 (28.9)
Weight, kg, median (IQR)	
Baseline (men)	93.4 (33)
Baseline (women)	79.3 (29)
Disease history	
Hypertension	39.6 (640)
Coronary heart disease	9.7 (157)
Diabetes	14.4 (232)
Hypothyroidism	25.1 (405)
Multiple sclerosis	3.5 (57)
Overweight	71.6 (1157)
Hypercholesterolemia	63.9 (1032)
Medications at baseline	
Blood pressure	33.6 (542)
Statin	22.1 (357)
Diabetes	12.0 (194)
Medication changes during program	
Reduced or stopped blood pressure medication, n/N (%)	469/542 (86.5)
Reduced or stopped diabetes medications, n/N (%)	176/194 (90.7)

As shown in Table 2, there were statistically significant decreases in cholesterol (total, HDL, and LDL), weight, systolic and diastolic blood pressure, blood glucose, creatinine, and blood urea nitrogen. The median (IQR) weight loss was 1.4 (1.8) kg. The median (IQR) decrease in total cholesterol was 22 (29) mg/dL. Patients with the highest baseline values for these markers showed the greatest improvements at day 7. For example, the median change in total cholesterol was -39 mg/dL for patients with a baseline value of ≥240 mg/dL but only -8 mg/dL for patients with a baseline value of <150 mg/dL. Systolic blood pressure dropped by 18 mm Hg in patients who had elevated systolic pressure at baseline but by only 4 mm Hg in patients whose systolic pressure was normal at baseline. The same was true for diastolic blood pressure (11 mm Hg drop compared to a 3 mm Hg drop). Even patients whose biomarkers were normal at baseline showed significant improvements at the end of the 7-day period on nearly every measure (except for patients with creatinine levels less than 1 mg/dL at baseline), although the greatest improvements were in the patients whose biomarkers were most abnormal at baseline.

**Table 2:** **Comparison of baseline and day 7 biomarker values (N = 1615)**

Variable	Baseline, median (IQR ^***a***^)	Day 7, median (IQR)	Change, median (IQR ^***b***^)	***P***-value
Weight, kg				
Overall	84.4 (31.4)	82.6 (30.4)	-1.4 (1.8)	<.001
Men	93.2 (32.2)	92.5 (30.8)	-1.5 (2.2)	<.001
Women	79.2 (29.0)	78.0 (28.5)	-1.4 (1.6)	<.001
Total cholesterol, mg/dL				
Overall	184 (56)	162 (49)	-22 (29)	<.001
< 150 at baseline	133 (20)	126 (127)	-8 (23)	<.001
150–179 at baseline	165 (16)	146 (25)	-20 (24)	<.001
180–209 at baseline	193 (14)	169 (25)	-25 (26)	<.001
210–239 at baseline	221 (15)	188 (28)	-34 (27)	<.001
≥ 240 at baseline	261 (31)	222 (43)	-39 (29)	<.001
HDL cholesterol, mg/dL (N = 1092)	49 (19)	40 (16)	-8 (8)	<.001
LDL cholesterol, mg/dL (N = 1091)	107 (49)	92 (41)	-16 (23)	<.001
Systolic blood pressure, mm Hg				
Overall	128 (26)	120 (23)	-8 (18)	<.001
Elevated (≥140) at baseline	150 (23)	136 (18)	-18 (18)	<.001
Normal (<140) at baseline	120 (18.5)	114 (20)	-4 (16)	<.001
Diastolic blood pressure, mm Hg				
Overall	80 (16)	74 (12)	-4 (10)	<.001
Elevated (≥90) at baseline	94 (10)	83 (12)	-11 (11)	<.001
Normal (<90) at baseline	76 (10)	70 (13)	-3 (10)	<.001
Triglycerides, mg/dL				
Overall	118 (89)	118 (86)	+2 (48)	.344
Normal (<150)	92 (49)	98 (53)	+8 (34)	<.001
Borderline high (150–199)	169 (22)	157.5 (73.5)	-11.5 (70.5)	.004
High (200–499)	247 (81)	216 (102)	-46 (100)	<.001
Very high (≥500)	569 (109)	360 (98)	-294 (131)	<.001
Glucose, mg/dL				
Overall	92 (19)	89 (15)	-3 (11)	<.001
< 100	88 (9)	86 (9)	-1 (7)	<.001
100 to 126	107 (10)	99 (13)	-8 (18)	<.001
> 126	156 (72)	149 (84)	-17 (45)	<.001
Blood urea nitrogen, mg/dL				
Overall	12 (5)	9 (4)	-3 (4)	<.001
< 10	8 (2)	7 (3)	-1 (2)	<.001
10 to 20	13 (4)	9 (3)	-4 (3)	<.001
> 20	23 (6)	14 (7)	-10 (5)	<.001
Creatinine, mg/dL				
Overall	0.8 (0.3)	0.8 (0.3)	0 (0.1)	<.001
< 1	0.8 (0.2)	0.8 (0.2)	0 (0.1)	.100
1 to 1.4	1.0 (0.2)	1.0 (0.1)	0 (0.1)	<.001
> 1.4	1.6 (0.3)	1.5 (0.3)	0.1 (0.2)	.002
10-year risk for hard ASCVD,% (N = 1092)	4.0 (9.0)	3.9 (9.0)	-1.0 (1.0)	<.001
Patients with elevated risk at baseline, percentage points (N = 403)	18.0 (17.5)	16.2 (16)	-2.0 (4.1)	<.001

^*a*^IQR = interquartile range.

^*b*^Notice that the change column is not the difference between the baseline and day 7 medians. The change score provided by the Wilcoxon signed rank test reflects the *paired* difference in the 2 scores.

Table 1 shows the number of patients taking medication at baseline and the number who reduced or discontinued medications during the program. Of those on medication upon entering the program, 86.5% of patients on blood pressure medications and 90.7% of patients on diabetes medications reduced their dosage or discontinued the medication entirely.

The 10-year risk of ASCVD for the full sample (where HDL cholesterol data were available for both baseline and day 7, N = 1092) experienced a median decrease of 1 percentage point (IQR = 1.0; p < .001). However, among patients whose risk was elevated (i.e., >7.5%) at baseline, the median decrease in 10-year risk at day 7 was 2.00 percentage points (IQR, 4.1; p < .001).

Table 3 shows that the percentage of patients whose blood pressure and total cholesterol went from elevated at baseline to normal at day 7 was much higher than the percentage of patients whose values went from normal to elevated in the same timeframe (p < .001, McNemar’s test).

**Table 3:** **Biomarker shifts from elevated to normal and vice versa from baseline to day 7**

Biomarker	Elevated at baseline and normal on day 7, no. (%)	Normal at baseline and elevated at day 7, no. (%)	P-value ^***a***^
Diastolic blood pressure	223 (13.8)	36 (2.2)	<.001
Systolic blood pressure	312 (19.3)	58 (3.6)	<.001
Total cholesterol	323 (20.0)	24 (1.5)	<.001

^*a*^McNemar’s test.

## Discussion

This study documents that a low-fat (≤10% of calories), high-fiber, high-carbohydrate (~80% of calories), vegan diet allows overweight patients to lose weight even if they eat enough food to feel completely satisfied, ad libitum. From eating such a diet for 7 days, the participants lost a median of 1.4 kg (3 pounds) and had significant improvements in blood pressure, blood lipids, and blood sugar. The patients who were the most overweight and had the most unfavorable biomarkers at baseline had the largest favorable responses. The improvements in blood pressure and blood sugar are even more remarkable when one considers that most of the patients discontinued their antihypertensive and antihyperglycemic medication at baseline.

This kind of dramatic, early success can be important for maintaining patients’ motivation to adhere to the diet. These rapid results could also motivate physicians to prescribe diet therapy before resorting to the pharmacy for helping their patients. The present study also provides a practical model for health promotion within the healthcare system: an intensive week-long residential educational program. The long-term effectiveness and cost-effectiveness of this approach for managing Western diseases, in comparison with the current approaches that focus on prescription medication and major surgery, deserve further study. We believe that this simple dietary approach can improve patients’ health and ultimately reduce healthcare costs.

The diet used in this study differs significantly from some conventional approaches to weight loss. Note, however, that the potential therapeutic value of plant-based diets has been mentioned in the American Diabetes Association’s clinical practice recommendations [16] as well as in a position paper issued by the American Dietetic Association [17]. The diet in this study was <10% fat by calorie, whereas the American Heart Association recommends that fat should account for 25% to 35% of calories [18]. Unlike many popular weight loss regimens, the diet used in this study does not use calorie counting or other portion control strategies for weight loss.

When conventional dietary approaches fail to reverse type 2 diabetes and cardiovascular disease, prescription medications are used to control the biomarkers of chronic disease. Unfortunately, the drug-induced improvements in biomarkers do not always translate into reduced mortality [19]. Paradoxically, the attempts to use prescription medications intensively to help people with type 2 diabetes achieve glycosylated hemoglobin levels similar to those found in healthy people have led to an increase in mortality as well as to weight gain and increases in episodes of hypoglycemia [20]. In contrast, a low-fat vegan diet improves biomarkers by removing the cause of Western diseases: the rich Western diet.

Uncontrolled studies like this one can provide valuable evidence in situations where randomized, blinded clinical trials have not been or cannot be performed [21]. This study provides valuable evidence of the efficacy of a lifestyle intervention: the maximum results that patients can achieve under ideal circumstances (i.e, restricted food choices). Nevertheless, this study has some significant limitations. One concern is that the moderate exercise allowed during the program could have contributed to the observed weight loss. Another concern is the timeframe, which was too short to show whether the improvements in biomarkers would be sustained or would ultimately translate into decreases in morbidity and mortality. However, longer studies of high-carbohydrate, plant-based diets in an outpatient setting have also shown promising results [7–14]. In contrast, prospective cohort studies have shown that low-carbohydrate diets increase all-cause mortality and the risk of cardiovascular events [22–24].

Perhaps the biggest limitation of the vegan diet studies comes from the selection of the participants. Subjects who choose to participate in these studies are willing to try a low-fat vegan diet to improve their health and/or appearance. Thus, they may be more likely than the general population to adhere to the diet.

In conclusion, in this retrospective study, we observed that an energy-unrestricted low-fat, starch-based, vegan diet consumed in a controlled environment for 7 days resulted in substantial favorable changes in commonly tested biomarkers that are used to predict future risks for cardiovascular disease and metabolic diseases. The results also suggest a model for a cost-effective therapy to offer patients commonly seen in medical practices today.

### Supporting data

The supporting data from this study will be available in a separate file for publication.

## Abbreviations

ASCVD: Atherosclerotic cardiovascular disease
HDL: High-density lipoprotein
IQR: Interquartile range
LDL: Low-density lipoprotein.
