# Erratum to: Effects of 7 days on an ad libitum low-fat vegan diet: the McDougall Program cohort

**DOI:** 10.1186/s12937-017-0234-9

**Published:** 2017-02-10

**Authors:** John McDougall, Laurie E. Thomas, Craig McDougall, Gavin Moloney, Bradley Saul, John S. Finnell, Kelly Richardson, Katelin Mae Petersen

**Affiliations:** 1 Dr. McDougall’s Health and Medical Center, PO Box 14039, Santa Rosa, CA 95402 USA; 2 Madison, NJ USA; 3 Healthy Living Program, Northwest Permanente, Beaverton, OR USA; 4 Department of Biostatistics, University of North Carolina, Chapel Hill, NC USA; 5 AOMA Graduate School of Integrative Medicine, Austin, TX USA; 6 Richardson Statistical Consulting, Iowa City, IA USA

## Erratum

After the publication of this article [1] it was noticed that in Table 2 in the second to last row the data needs to be changed. Please see the updated Table 2 below.

**Table 2:** Comparison of baseline and day 7 biomarker values (N=1615)

Variable	Baseline, median (IQR^*a*^)	Day 7, median (IQR)	Change, median (IQR^*b*^)	*P*-value
Weight, kg				
Overall	84.4 (31.4)	82.6 (30.4)	-1.4 (1.8)	<.001
Men	93.2 (32.2)	92.5 (30.8)	-1.5 (2.2)	<.001
Women	79.2 (29.0)	78.0 (28.5)	-1.4 (1.6)	<.001
Total cholesterol, mg/dL				
Overall	184 (56)	162 (49)	-22 (29)	<.001
<150 at baseline	133 (20)	126 (127)	-8 (23)	<.001
150-179 at baseline	165 (16)	146 (25)	-20 (24)	<.001
180-209 at baseline	193 (14)	169 (25)	-25 (26)	<.001
210-239 at baseline	221 (15)	188 (28)	-34 (27)	<.001
≥240 at baseline	261 (31)	222 (43)	-39 (29)	<.001
HDL cholesterol, mg/dL (N=1092)	49 (19)	40 (16)	-8 (8)	<.001
LDL cholesterol, mg/dL (N=1091)	107 (49)	92 (41)	-16 (23)	<.001
Systolic blood pressure, mm Hg				
Overall	128 (26)	120 (23)	-8 (18)	<.001
Elevated (≥140) at baseline	150 (23)	136 (18)	-18 (18)	<.001
Normal (<140) at baseline	120 (18.5)	114 (20)	-4 (16)	<.001
Diastolic blood pressure, mm Hg				
Overall	80 (16)	74 (12)	-4 (10)	<.001
Elevated (≥90) at baseline	94 (10)	83 (12)	-11 (11)	<.001
Normal (<90) at baseline	76 (10)	70 (13)	-3 (10)	<.001
Triglycerides, mg/dL				
Overall	118 (89)	118 (86)	+2 (48)	.344
Normal (<150)	92 (49)	98 (53)	+8 (34)	<.001
Borderline high (150-199)	169 (22)	157.5 (73.5)	-11.5 (70.5)	.004
High (200-499)	247 (81)	216 (102)	-46 (100)	<.001
Very high (≥500)	569 (109)	360 (98)	-294 (131)	<.001
Glucose, mg/dL				
Overall	92 (19)	89 (15)	-3 (11)	<.001
<100	88 (9)	86 (9)	-1 (7)	<.001
100 to 126	107 (10)	99 (13)	-8 (18)	<.001
>126	156 (72)	149 (84)	-17 (45)	<.001
Blood urea nitrogen, mg/dL				
Overall	12 (5)	9 (4)	-3 (4)	<.001
<10	8 (2)	7 (3)	-1 (2)	<.001
10 to 20	13 (4)	9 (3)	-4 (3)	<.001
>20	23 (6)	14 (7)	-10 (5)	<.001
Creatinine, mg/dL				
Overall	0.8 (0.3)	0.8 (0.3)	0 (0.1)	<.001
<1	0.8 (0.2)	0.8 (0.2)	0 (0.1)	0.100
1 to 1.4	1.0 (0.2)	1.0 (0.1)	0 (0.1)	<.001
>1.4	1.6 (0.3)	1.5 (0.3)	0.1 (0.2)	0.002
10-year risk for hard ASCVD, % (N=1092)	4.4 (11.4)	3.9 (9.9)	-0.2 (1.6)	<.001
Patients with elevated risk at baseline, percentage points (N=403)	18.0 (17.5)	16.2 (16)	-2.0 (4.1)	<.001

^*a*^IQR = interquartile range

^*b*^Notice that the change column is not the difference between the baseline and day 7 medians. The change score provided by the Wilcoxon signed rank test reflects the *paired* difference in the 2 scores
